# Designing Studies to Inform Tobacco Harm Reduction: Learnings From an Oral Nicotine Pouch Actual Use Pilot Study

**DOI:** 10.2196/37573

**Published:** 2022-08-19

**Authors:** Chris Campbell, Michael Feehan, Claudia Kanitscheider, Patrudu S Makena, Jenny Cai, Sarah A Baxter

**Affiliations:** 1 RAI Services Company Winston-Salem, NC United States; 2 Cerner Enviza, an Oracle company Kansas City, MO United States; 3 Department of Ophthalmology, Ross Eye Institute Jacobs School of Medicine and Biomedical Sciences State University of New York at Buffalo Buffalo, NY United States; 4 Department of Population Health Sciences University of Utah School of Medicine Salt Lake City, UT United States; 5 Cerner Enviza, an Oracle company Munich Germany

**Keywords:** harm reduction, pilot, nicotine pouch, actual use, electronic diary, smartphone, survey, combustible cigarette, smoking reduction, remote monitoring

## Abstract

**Background:**

Introduction of new tobacco products in the United States, including those that may be lower on the risk continuum than traditional combustible cigarettes, requires premarket authorization by the US Food and Drug Administration and information on the potential impact of the products on consumer behaviors. Efficient recruitment and data capture processes are needed to collect relevant information in a near-to-real-world environment.

**Objective:**

The aim of this pilot study was to develop and test a protocol for an actual use study of a new tobacco product. The product included in this study was a commercially available oral nicotine pouch. Through the process of study design and execution, learnings were garnered to inform the design, execution, analysis, and report writing of future full-scale actual use studies with tobacco products.

**Methods:**

A small sample (n=100) of healthy adult daily smokers of 7 or more cigarettes per day were recruited to participate in an 8-week prospective observational study conducted at 4 geographically dispersed sites in the United States. A smartphone-based customized electronic diary (eDiary) was employed to capture daily tobacco product use, including 1 week of baseline smoking and 6 weeks during which participants were provided with oral nicotine pouches for use as desired.

**Results:**

Online screening procedures with follow-up telephone interviews and on-site enrollment were successfully implemented. Of 100 participants, 97 completed the study, with more than half (59/99, 60%) identifying as dual- or poly-users of cigarettes and other types of tobacco products at baseline. There was more than 90% (91-93/99, 92%-94%) compliance with daily eDiary reporting, and the majority (92/99, 93%) of participants expressed satisfaction with the study processes. Product use data from the eDiary indicated that after an initial period of trial use, pouches per day increased among those continuing to use the products, while per day average cigarette consumption decreased for 82% (79/97) of all study participants. At the end of the week 6, 16% (15/97) of participants had reduced their cigarette consumption by more than half.

**Conclusions:**

The design of this study, including recruiting, enrollment, eDiary use, and oversight, was successfully implemented through the application of a detailed protocol, a user-friendly eDiary, electronically administered questionnaires, and remote monitoring procedures. High-resolution information was obtained on prospective changes in tobacco product use patterns in the context of availability of a new tobacco product. Future, larger actual use studies will provide important evidence supporting the role that alternatives to combustible cigarettes may play in smoking reduction and/or cessation and lowering the population health burden of tobacco and nicotine-containing products.

## Introduction

Combustion-related toxicants drive the adverse health effects associated with cigarette smoking, including cardiovascular disease, respiratory disease, and cancer, among others [1]. The American Cancer Society indicates that “smoking is by far the leading risk factor for lung cancer. About 80% of lung cancer deaths are thought to result from smoking” [2]. To reduce the harm associated with smoking, alternatives to combustible cigarettes that fall lower on the toxicant exposure and health risk continuum are increasingly available [3,4]. These can include heated tobacco products, electronic nicotine delivery systems, smokeless tobacco products, and oral nicotine pouches (ONPs). Similar to smokeless products, ONPs are placed in the mouth for use. ONPs contain nicotine and flavorants but no tobacco leaf, resulting in much lower toxicant levels compared to other combusted and noncombusted tobacco products. Thus, ONPs are anticipated to present fewer potential health risks to users [5,6].

Tobacco products such as ONPs are regulated by the US Food and Drug Administration (FDA) and require authorization from the Center for Tobacco Products before they can be sold in the United States [7]. Manufacturers must submit premarket tobacco applications with sufficient information for the FDA to determine whether their marketing is appropriate for the protection of public health [8]. The Center for Tobacco Products requires information to assess the potential impact of a new product on current tobacco user behaviors, including who uses the product, how the product is used, and the effects of its use on the use of other tobacco products. Various study designs can be employed to provide this information, including randomized clinical trials, longitudinal cohort studies, and actual use studies (AUS) [3,9-15].

Unlike in a randomized clinical trial with managed interventional arms, in the prevailing model of tobacco AUS, smokers (the intended user) are sufficiently supplied with study products and remain free to use or not use the product at their discretion. Research participants are followed over varying time periods to capture daily product use patterns, including use of the new product and any consequent changes from their baseline tobacco consumption patterns. An AUS can also provide information on whether tobacco users revert to using their usual tobacco products after initiating use of the new product, subjective experiences to inform use transition patterns, and whether users engage in product misuse. To date, few AUS findings are in the published literature. Two industry-sponsored studies have been published that assess the use of novel tobacco products [10,11]. In the first, over 1000 US smokers were provided a nonmarketed heated tobacco product for use over a 6-week period and recorded their tobacco product use using a daily electronic diary (eDiary) [10]. A similarly sized AUS was conducted to assess the use of a marketed ONP among adult US smokers and smokeless tobacco product users [11]. End points included complete substitution of combustible cigarette by the new products and reductions in levels of combustible cigarette use.

Collecting the required information on nonmarketed products is challenging due to the requirement of obtaining authorization for research use of new tobacco and nicotine-containing products (TNP), determining the appropriate sample population of who to include in studies (ie, current smokers or dual users of cigarettes and other tobacco products), and the cost of studies with large numbers of participants. In designing appropriate studies with large samples, it is beneficial to trial and test procedures to maximize efficiency around factors such as recruiting, optimizing the logistics around distributing study products (accommodating regulatory restrictions), and the establishing procedures to ensure capture of reliable use data on a frequent (at least daily) basis. Therefore, we conducted a pilot study with 100 adult smokers to inform the design, execution, analysis, and report writing of future full-scale AUSs.

## Methods

### Study Design

**Figure 1:**
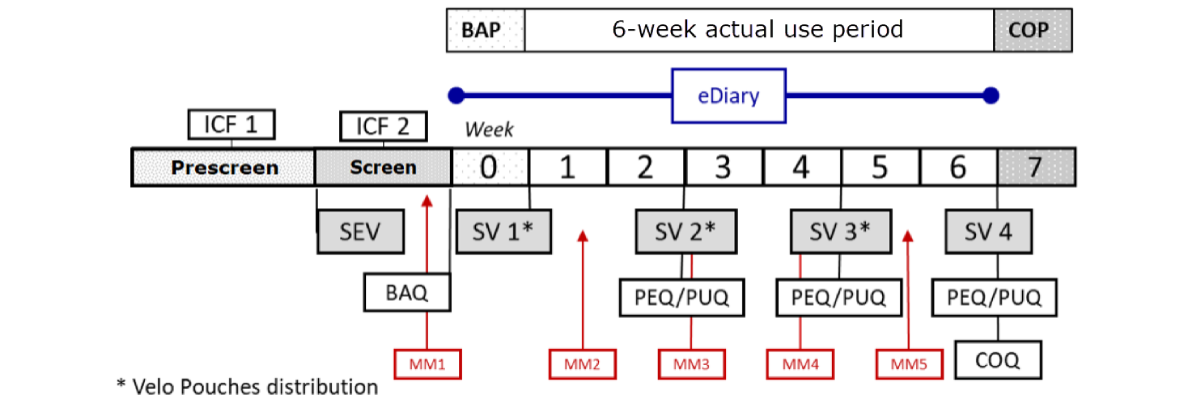
Overall study design. BAP: baseline assessment period; COP: close out period; ICF: informed consent form; SEV: screening and enrollment visit; SV: site visit; BAQ: baseline assessment questionnaire; PEQ: product experience questionnaires; PUQ: product use questionnaires; MM: marketing material; COQ: close out questionnaire.

This was a pilot multisite, open-label, 8-week, prospective observational study (Figure 1), conducted between September 25 and December 31, 2020, at 4 sites geographically dispersed across the United States. The primary objective was to describe the patterns of use for the ONPs and combustible cigarette over a 6-week actual use period (AUP) using a self-reported daily eDiary.

### Study Products

The study products were 2 oral nicotine pouches: Velo Pouch Mint (4 mg nicotine) and Velo Pouch Citrus (4 mg nicotine, Modoral Brands Inc). Participants could select which flavors they received and could move between flavors as desired. Originally supplied at site visit 1, the product was resupplied at follow-up site visits. Detailed product accountability logs were kept by site staff.

### Study Population

A total of 100 generally healthy US adults (aged 21 years or older) who were daily menthol and/or nonmenthol cigarette smokers of 7+ cigarettes per day (CPD) were recruited from nationally representative consumer databases of individuals agreeing to be contacted for market research studies.

The sample for this pilot study of 100 was considered adequate to develop and test procedures across multiple sites, with a reasonable number of participants per site (n=25) to allow for monitoring of the staff training, recruitment, and product distribution processes, across geographies. The sample size was not determined by any a priori formal sample size calculation.

Inclusion criteria included no intention of quitting tobacco use during the next 8 weeks, no participation in tobacco research studies in the past 3 months, and ability to complete all surveys in English. Anyone who used Velo Pouches currently or in the past; was pregnant, breastfeeding, or planning to become pregnant; or did not agree to restrict Velo Pouch use to only the products supplied in the study was excluded from participating.

### Procedures

The overall study design and notable events are provided Figure 1. Candidate participants were identified using a 2-stage screening and informed consent process. During the first stage (prescreening), a computer-assisted telephone interview identified interested and eligible candidate participants. These candidates gave verbal consent for further screening at the on-site screening and enrollment visit, where eligibility was reconfirmed and age was verified with a photo identification. Prior to signing the second informed consent form, eligible candidates reviewed product information and physical examples of the study products. Those who indicated an intention to use the products at least once during the 6-week study underwent the second informed consent process before any protocol-specific procedures were carried out.

The second informed consent form explained the full nature of the study, including the optional use of study products, use of a daily eDiary, frequency of repeat visits to the sites for interviews and questionnaire completion, description of the study products, and expected experiences of their use. In addition to having the participants read the informed consent forms themselves, trained site staff explained the research study to the research participants and answered any questions that arose. A verbal explanation was provided in terms suited to their comprehension of the purposes, procedures, and potential risks of the study and of their rights as research participants. Restrictions and requirements of the study were also explained to the participants. Participants were informed that participation was voluntary and they could withdraw from the study at any time, without prejudice. Participants confirmed their willingness to be in the study using via electronic signature, were enrolled in the study, and completed the baseline assessment questionnaire (Multimedia Appendix 1) prior to leaving the site and beginning the baseline assessment period. The baseline assessment questionnaire captured additional demographic information (eg, education, occupational status, income), past 30-day TNP use, and supplemental questions related to cigarette dependence.

During the baseline assessment period (week 0), participants recorded their daily tobacco product use in the eDiary as described below. Following the baseline assessment period, participants participated in a 6-week AUP in which they were allowed to use the nicotine pouch study products and all TNP use was recorded daily. Participants were scheduled for in-person site visits 5 times during the study as noted in Figure 1 (screening and enrollment visit and site visits 1 to 4). Participants received reminders prior to the day of each follow-up visit by email (1-3 days prior) and by telephone (1 day prior).

At site visits 1 through 3, study products were provided at no cost for use as desired. At site visits 2 through 4, participants were interviewed to review their prior period’s eDiary compliance and share their experience with and use of the ONPs via a product experience questionnaire (Multimedia Appendix 2) and product use questionnaire (Multimedia Appendix 3). Given the study was fielded during the COVID-19 pandemic, appropriate mitigation procedures were in place (temperature testing, social distancing, masking, etc) to ensure participant and staff safety. At the close of the AUP on site visit 4, participants returned all unused product and were asked about their satisfaction with study participation via a closeout questionnaire (Multimedia Appendix 4). The 1-week closeout period allowed participants to communicate any adverse events (AEs) or ask additional study questions.

### eDiary

Due to the high frequency of daily use over an extended period of time, participants used an eDiary installed on their personal smart devices to record their daily combustible cigarette and other TNP use rather than a paper diary. This practice decreased the overall study burden on participants, reduced potential sources of error when transferring data from a paper diary to a study database, and allowed for familiarity and ease of use of the device by participants. Those who did not own smartphones or whose own devices were incompatible with the eDiary app were provided provisioned devices and limited data plans for the duration of the study. This ensured that barriers to enrollment were not created for those of limited means or lower socioeconomic status. The eDiary was an easy-to-use third-party app slightly modified by its developers for use in this study and previously used in other published health-related studies [16-18].

The app was programmed to capture daily TNP use during a set 6-hour time window every evening. Four unique electronic marketing executions were delivered via the eDiary app, one approximately every 7 to 9 days, to simulate advertising exposure as in the real world. Participants received daily notifications that their eDiary was open for completion and compliance was monitored by site staff through an online portal including sites sending additional reminders or phone calls for any data missing for the prior 2 days.

Electronic patient-reported outcome best practices were followed in its design [19]. All information collected via the app was held by the developer on a restricted access secure database in compliance with the appropriate data protection regulations in the United States. Any personal information such as email addresses and IP addresses were kept separately and securely and not linked to any other data. Participants were assigned a unique identification number to link the data with the questionnaire data in the EDC and only pseudoanonymized, individual-level data were processed for the purposes of the study.

### Data Management and Analysis

All eDiary and questionnaire data were collected by an FDA 21 Code of Federal Regulations Part 11 compliant electronic data capture system [20].

Data sets were created and exported for analysis according to FDA standards. Although the small number of participants in this pilot study precluded detailed substantive analyses, a comprehensive statistical analysis plan and study report were prepared according to International Council for Harmonisation of Technical Requirements for Pharmaceuticals for Human Use guidelines to prepare for and identify additional requirements that would be used for planning larger studies [21]. All analysis were descriptive by design, with no a priori statistical hypotheses and no statistical testing or multivariate modeling. The statistical evaluation was performed with the software package SAS (release 9.4, SAS Institute Inc).

### Ethics Approval

#### Oversight

The final protocol, informed consent forms, and all pertinent study documents were reviewed and approved by the Sterling Institutional Review Board (reference number: 8229) prior to participant recruitment and any study procedures. The study was conducted using standard operating procedures adopted by Cerner Enviza, an Oracle company, and was conducted under the rubric of good epidemiological practice [22].

#### Monitoring and AEs

A telephone hotline was available throughout the study for participants to report any pregnancies, product complaints, or AEs associated with the study products. A separate monitoring team ensured adherence to the protocol and a detailed procedures manual which included interview and product accountability guidelines. In-person monitoring was intended to be conducted on-site but due to COVID-19 was converted to a remote monitoring process which used streaming technology to observe participant interviews and confirm site compliance. As an observational study with commercially available tobacco products and no clinical end points, this study was not submitted to any publicly available trial registry.

#### Compensation

Participants received industry-standard prorated compensation at each site visit for time spent on completing the daily eDiary and attending the site visits. The compensation schedule was designed to maximize return to sites for periodic visits and completion of the study, so honoraria were distributed at key milestones. The compensation did not depend upon the use of any tobacco or nicotine-containing products, including their own combustible cigarettes or study-provided nicotine pouches.

## Results

### Recruitment

This pilot study was successful in terms of designing and evaluating all the procedures and processes necessary for a full-scale AUS. A total of 3690 candidate participants were screened online, 223 were interviewed by telephone, and 137 met all criteria, including preliminary interest in the study product, and were scheduled for a screening and enrollment visit. There was a 20% no show rate for the screening and enrollment visit, and a screen fail rate of 7% at the screening and enrollment visit.

Once the target sample size of 100 was achieved, screening was discontinued. The predominant reasons for not qualifying for the study were not being a daily smoker of the minimum of 7 CPD, having previously used Velo Pouches, no interest in using the study product, and currently quitting or planning to quit all TNP use in the next 8 weeks.

### Participant Demographics

Participant demographic characteristics at enrollment are presented in Table 1 (n=100). All participants in the final analysis set (n=99 after exclusion of 1 participant who did not fulfill criteria of using at least 1 pouch) started regular smoking more than 12 months prior to study entry. Participants’ self-reported CPD mean was 14.6 (SD 5.72) during the 6 months prior to study entry, and just over half (59/99, 60%) of participants reported also using other types of tobacco products (eg, electronic nicotine delivery systems, smokeless tobacco) within the past 30 days. Approximately half (49/99, 50%) were classified as having low nicotine dependence based on a total score of 4 or less on the Fagerström Test for Nicotine Dependence [23].

Over the study period there was very low attrition (3%: 1 withdrawal, 2 lost to follow-up). Attendance at each site visit was high, with only 3 participants missing a scheduled site visit 2 or 3.

**Table 1:** Participant demographic characteristics at enrollment (n=100).

Characteristic		Value
Age (years), mean (SD)		48.1 (11.03)
**Age category (years), n (%)**		
	21-48	48 (48.0)
	>49	52 (52.0)
**Gender, n (%)**		
	Male	56 (56.0)
	Female	44 (44.0)
	Nonbinary	0 (0.00)
**Race, n (%)**		
	White	61 (61.0)
	Black or African American	35 (35.0)
	All other	4 (4.0)
**Ethnicity, n (%)**		
	Hispanic or Latino	6 (6.0)
	Not Hispanic or Latino	94 (94.0)
**Education, n (%)**		
	Some high school	2 (2.0)
	High school degree or equivalent	58 (58.0)
	College graduate	40 (40.0)
**Income ($), n (%)**		
	<39,999	31 (31.0)
	40,000-79,999	50 (50.0)
	>80,000	19 (19.0)
**Employment, n (%)**		
	Working now	70 (70.0)
	Not working	30 (30.0)

### eDiary Experience and Compliance

The eDiary was well received with very high participant compliance. More than 90% (91-93/99, 92%-94%) of participants were fully compliant with daily eDiary entries, and 97% (95-98/99, 96%-99%) reported their product use at least 5 days during each week of the AUP.

Only a small number of participants (15/99, 15%) required reminder calls (a total of 19 calls) if they had not completed the diary at the end of the day.

In addition to the functional success of study procedures and processes, the study was viewed positively by participants with 93% (92/99) satisfied with the experience and 95% (94/99) were being likely to recommend the study to others (87/99, 88% very likely; 7/99, 7% likely).

Some participants suggested improvements for future studies such as providing more flexibility around time frames to complete the eDiary (9 mentions). Others indicated a preference for a broader range of product options (flavors/nicotine levels; 5 mentions), and for remote interviewing (3 mentions).

The product use questionnaire showed that the majority of use was as described on the product packaging and communicated to participants during the informed consent process. In total, only 4 participants reported using Velo Pouches in any way other than instructed and none were associated with any AE report. One participant sucked the pouch, one moistened the pouch with water, one moistened the pouch on the tongue, and one participant used two pouches at the same time. Further, 15 participants used the product at the same time as another TNP, two accidentally swallowed the pouch, and 38 reported spitting out saliva while using the product. No safety issues emerged during the study, and no AE reports required adjudication.

### Product Use

**Figure 2:**
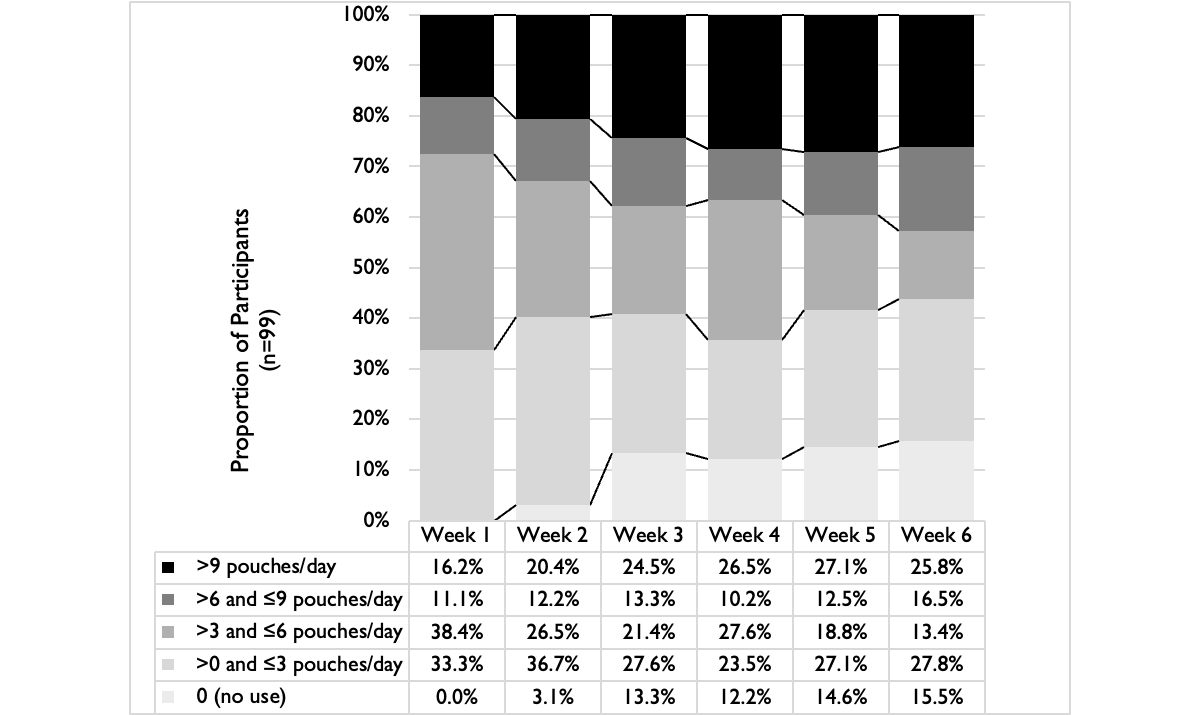
Changes in frequency of pouch use/day over 6-week actual use study.

In the first week of the AUS (following the baseline assessment period), all participants tried the study ONPs (Figure 2). Over time, the proportion of participants using ONPs decreased (from 0% reporting no use in week 1 to 15.5% [15/97] in week 6). Among those who continued using the ONPs, the average pouches per day increased (Figure 2, darker bars). Whereas the proportion of participants using between 1 and 6 pouches per day decreased between week 1 and week 6 (71/99, 72%, to 40/97, 41%), the proportion using 7 or more pouches per day increased from 27% (27/99) in week 1 to 42% (41/97) in week 6.

**Figure 3:**
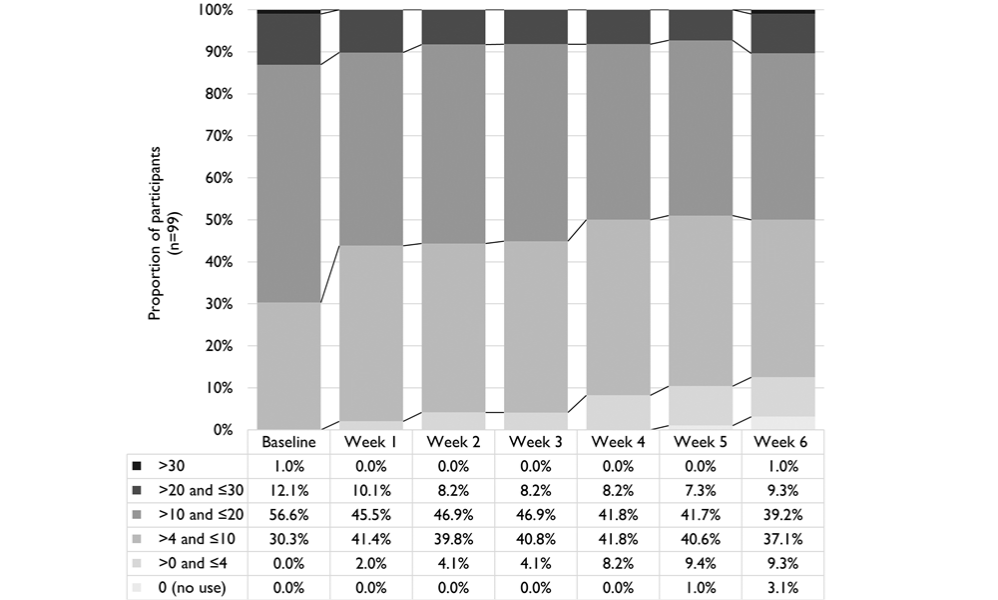
Changes in cigarettes per day use from baseline over 6-week actual use study.

Of interest for potential tobacco harm reduction, combustible cigarette use decreased over the study period for 82% (79/97) of the study participants (see Figure 3). At week 6, approximately 16% (15/97) of participants reduced their cigarette consumption by more than 50%, 18% (17/97) reduced their CPD between 30% and 50%, and almost half (47/97, 49%) reduced their CPD between 1% and 30%.

## Discussion

### Principal Findings

The success of this pilot study in terms of methods and procedures relied on a detailed protocol, user friendly eDiary, and electronically administered questionnaires to capture study product and TNP use and other information about product use patterns. Experience during the pilot study execution highlighted some considerations to be incorporated in future AUS designs.

#### Selection Criteria

Because of the high level of dual use, particularly with electronic nicotine delivery systems, at baseline, future studies should consider maintaining a broad definition of eligible participants to encompass a real-world demography. Traditional studies on tobacco harm reduction have tended to use only daily combustible cigarette use as an entry criterion [24-26]. A definition of regular use may be more appropriate as product use behaviors are changing with increasing availability and acceptance of other forms of TNP [27,28].

Similarly, many daily smokers in the candidate pool did not meet the 7 CPD threshold. With the rise of combustible cigarette alternatives and an overall decline in combustible cigarette use, lower thresholds for study entry may be warranted to reflect the real-world behaviors of those likely to use the new products in the future, including for those looking to completely supplant combustible cigarette use [29]. Optimally, participants should have sufficient combustible cigarette daily use to detect a change in combustible cigarette use behaviors over the time of the study.

Candidate participants were excluded if they were quitting or intending to quit all TNP use. Future AUS may relax this criterion and allow the inclusion of those intending to quit combustible cigarette but continue use of other TNP. This would allow for an investigation of differences in product use and changes in cigarette smoking among smokers who were not immediately interested in quitting all TNP [30].

#### Recruitment

Recruitment of the 100 planned pilot study participants via market research sites was successful. A larger AUS powered to achieve narrow confidence intervals around product adoption or CPD reductions may necessitate using additional recruitment strategies to boost or augment the number of combustible cigarette smokers in their databases and to increase diversity of enrollees. For example, a multimodal recruiting strategy may be needed to increase representation of younger adult smokers [31,32]. This pilot was conducted in only 4 sites and recruited a relatively large proportion of African American participants, while the number of those of other races and ethnicities was low. Identifying sites with higher populations of other important subgroups will also be helpful in expanding demographic diversity for future studies.

#### Product Use

Since there was no sampling of the product during the enrollment or baseline period, participants first opportunity to try the product was during week 1 of the AUP. Nearly all participants used pouches in the first 2 weeks. The majority rated their liking of the product as in between. By week 6, the number with strong positive reactions to the product increased, and the data suggest that those who did not like the product after trial discontinued use whereas those who liked the product increased their use. Furthermore, product use other than as directed was infrequent and did not suggest any significant modifications to procedures or participant instructions in future studies.

Although the number of enrolled participants was small and precludes a detailed statistical analysis and interpretation, the results of ONP uptake and CPD reduction provide a directional indication of potential product use in a real-world setting that can be tested in follow-up studies with larger sample sizes.

### Comparison With Prior Work

The pilot results were in a positive direction for the potential of ONP to supplant combustible cigarette use, in line with preliminary trends seen in a recent ONP AUS with a larger sample size [11]. The study attrition was low, and eDiary compliance in our study was high as compared to other reported studies with shorter time frames [33]. Low attrition may be attributable to the compensation level and in-person payments being spread over the entire 8-week study. High eDiary compliance could have resulted from reinforced attention to the eDiary via intermittent marketing material delivery, regular reminders to complete the eDiary, and the close monitoring of the eDiary data by site staff. Once per day eDiary entries, rather than multiple entries throughout a given day, also helped to reduce the protocol burden on participants and improve compliance [34]. Given the eDiary success, one key learning from this study was to expand the technology used to manage the study. In this study, 2 systems were used for data capture—a central database for eDiary data and individual site spreadsheets for product dispensation and return. Integrating these data streams into a single portal-based system will improve efficiency in managing products, reconciling product use data, and requesting product-specific feedback so that item responses can be captured for only the products that have been distributed to a particular participant.

Consistent with moves toward more decentralized clinical trials [35,36], the pivot to remote monitoring due to COVID-19 was successful for this study and will be incorporated in future studies. Videoconferencing tools, electronic data capture, and electronic signatures allowed for more efficient and timely communication, expanded sponsor participation in on-site activities, and reduced travel-related costs and off-site supervision of participant interviews by monitoring staff.

### Limitations

The main limitation of this actual use pilot study is its small size. However, the purpose of the study was to determine the logistic feasibility of conducting a full-scale study. Future studies with much larger sample sizes will have the power to test for explicit associations between combustible cigarette use and study product use and the variation by subgroups of interest (eg, by demographics or smoking history). As with all AUSs, general limitations exist. Participants were all incentivized to participate and received study product at no charge, both of which can limit generalizability.

All data collected were via participants’ self-report in a real-world setting. Future AUSs could consider including measurement of change in any proximal biomarkers that could reflect more distal health outcomes. Collecting relevant biosamples (blood, urine, respiratory output) at the start and end of an AUP (especially if longer than a 6-week duration) could provide early indicators of improved health function among those who reduce their cigarette consumption though use of study products. Since participants returned to each site for product fulfillment, a degree of social desirability bias is possible if participants believed the interviewers expected to see high levels of study product use. The informed consent form at enrollment explained the optional nature of using study product, which was reinforced in staff training and ongoing monitoring interviews. The eDiary data were consistently captured, regardless of the level of study product use.

All questionnaires and the eDiary relied on participants’ recall of their TNP use, which can be affected by time since use, environmental considerations, and other factors outside the control of the investigators. With this in mind, participants were given a short, 2-day grace period to enter product use information. Including this small window around diary entries limited recall errors while improving participant compliance. Other research has demonstrated high degree of congruity between eDiary logs and actual combustible cigarette use [37]. Since all TNP uses were captured, the risk of differential recall for cigarettes versus pouches would be minimal and unlikely to be biasing. The small number of participants who required reminders to complete the diary the study would preclude meaningful analyses to gauge the potential impact of any delayed data entry bias, which could certainly be examined in a full larger AUS.

In the closeout questionnaire, a small number of participants made suggestions for future studies. These suggestions can be considered in the design of future studies, although there are limitations to their applicability. Participants suggested more product choices, which will be determined by future research needs and market changes. Some suggested expanding the time frame of eDiary completion, which would necessitate a trade-off between satisfying individual preference flexibility against the minimization of programming and analytic complexity. Finally, a few suggested the preference for being interviewed remotely. This practice is now much more common in accommodation of the COVID-19 pandemic and would be very helpful for several reasons. It could greatly expand the geographical and demographic diversity of participants, who could be interviewed at time convenient to them without needing transportation, childcare, or time away from work, and would allow for increased efficiency and consistency across all sites with the use of remote interviewers. The challenge remains that any AUS would still require close product accountability and means to get products into the hands of age-verified respondents (and return of unused products), in a regulatory environment where there can be significant legal and logistical restrictions to remote distribution of study products.

### Conclusions

This pilot (and other research studies) provides an opportunity to assess the impact of product introduction on existing product use patterns. The initial trends from this work provide evidence that the availability and use of this alternative TNP may be associated with combustible cigarette reduction or smoking cessation use and thus has the potential to positively impact public health. The execution of this pilot study with very low participant attrition, successful daily eDiary data collection, and strong participant satisfaction indicate a high likelihood of success for future fuller AUS with much larger samples and broader geographical distribution. If regulatory and legal challenges can be overcome and alternative study product distribution methods become available (eliminating attendance at sites in person), such studies will be able to attract a broader demographic mix, especially among groups of participants with barriers to study participation because of logistical difficulties. Finally, as we look to the future and the ultimate public health goal of reducing the deleterious impact of cigarette smoking on the public health, incorporating biomarkers of exposure/biological effect and biomarkers of potential harm in AUSs could provide early indicators of ultimate improved health outcomes.

## Appendix

Multimedia Appendix 1 Baseline Assessment Questionnaire items.

Multimedia Appendix 2 Product Experience Questionnaire.

Multimedia Appendix 3 Product Use Questionnaire.

Multimedia Appendix 4 Closeout Questionnaire.

## Abbreviations

AE: adverse event
AUP: actual use period
AUS: actual use study
CPD: cigarettes per day
eDiary: electronic diary
FDA: US Food and Drug Administration
ONP: oral nicotine pouch
TNP: tobacco or nicotine-containing product
