# Supplementary material for: Designing Studies to Inform Tobacco Harm Reduction: Learnings From an Oral Nicotine Pouch Actual Use Pilot Study
Source: JMIR Form Res. 2022 Aug 19;6(8):e37573. doi: 10.2196/37573 (PMC9440415; doi:10.2196/37573)
Supplement: Multimedia Appendix 1 [file formative_v6i8e37573_app1.docx]

Multimedia Appendix 1. Baseline Assessment Questionnaire items.

|  | Item |
| --- | --- |
| A. | How soon after you wake up do you smoke your first cigarette? (Within 5 minutes, 6 to 30 minutes, 31 to 60 minutes, After 60 minutes) |
| B. | Do you find it difficult to refrain from smoking in places where it is forbidden, for example in church, at the library, in theaters? (Yes, No) |
| C. | Which cigarette would you hate most to give up? (The first one in the morning, Any other) |
| D. | How many cigarettes per day do you smoke? (10 or less, 11 to 20, 21 to 30, 31 or more) |
| E. | Do you smoke more frequently during the first hours after waking than during the rest of the day? (Yes, No) |
| F | Do you smoke if you are so ill that you are in bed most of the day? (Yes, No) |
| G. | What is your highest education level achieved? (Grade school (grades 1-8), High school (grades 9-11), High school graduate (grade 12) or GED, Technical school, Some college, College graduate, Graduate school, Don’t know/Unsure, Decline to answer) |
| H. | What is your current occupational status? (Working now; Only temporarily laid off, sick leave or maternity leave; Looking for work, unemployed; Retired; Disabled, permanently or temporarily; Homemaker, keep housing; Student; Other; Don’t know/Unsure; Decline to answer) |
| I. | What is your yearly household income? (Less than $20,000, $20,000 to $39,999, $40,000 to $59,999, $60,000 to $79,999, $80,000 to $99,999, $100,000 to $119,999, $120,000 or more, Don’t know/Unsure, Decline to answer) |
| J. | In addition to cigarettes, which of the following tobacco or nicotine containing products have you used in the past 30 days? (Filtered Cigars, Loose Moist Snuff, Pouched Moist Snuff, Snus, Chewing Tobacco, Dry Snuff, Dissolvable Tobacco / Nicotine Lozenges, Electronic Cigarettes, Roll your own cigarettes, Nicotine replacement therapies, Other, None of the above) |
| K. | How frequently have you used? (Every day, Frequently (more than 5 times in the last 30 days), Infrequently (5 or fewer times in the last 30 days)) |
