# Supplementary material for: Designing Studies to Inform Tobacco Harm Reduction: Learnings From an Oral Nicotine Pouch Actual Use Pilot Study
Source: JMIR Form Res. 2022 Aug 19;6(8):e37573. doi: 10.2196/37573 (PMC9440415; doi:10.2196/37573)
Supplement: Multimedia Appendix 2 [file formative_v6i8e37573_app2.docx]

Multimedia Appendix 2. Product Experience Questionnaire.

|  | Item | Scale |
| --- | --- | --- |
| A. | At this moment, what is your overall liking of [Product]? | 0-10 Likert Scale (0-strongly dislike, 5-neither like nor dislike, 10-strongly like)  I have not used [Product] in the past two weeks |
| B. | At this moment, what is your overall liking of [Product]? | 0-10 Likert Scale (0-strongly dislike, 5-neither like nor dislike, 10-strongly like)  I have not used [Product] in the past two weeks |
