# Supplementary material for: Designing Studies to Inform Tobacco Harm Reduction: Learnings From an Oral Nicotine Pouch Actual Use Pilot Study
Source: JMIR Form Res. 2022 Aug 19;6(8):e37573. doi: 10.2196/37573 (PMC9440415; doi:10.2196/37573)
Supplement: Multimedia Appendix 3 [file formative_v6i8e37573_app3.docx]

Multimedia Appendix 3. Product Use Questionnaire.

|  | Item |
| --- | --- |
| A. | In the past two weeks, have you used [Product] in any way other than how you were instructed to use them? (Yes/No) |
| B. | Please describe the way you used [Product]? (Verbatim) |
| C. | How many times in the past two weeks have you used [Product] in a way other than how you were instructed to use them? (# of Times) |
| D. | In the past two weeks, have you used [Product] at the exact same time that you were using another tobacco- or nicotine-containing product? (Yes/No) |
| E. | How many times did you use [Product] at the exact same time you were using other tobacco and nicotine products? (# of Times) |
| F | What type of tobacco or nicotine product did you use at the same time you were using Velo Pouch?” (Read list) (Select all that apply) (Cigarettes, Vaping Device, Loose Smokeless Tobacco Product, Pouched Smokeless Tobacco Product, other Nicotine Pouch, Nicotine Lozenge, Other Tobacco or Nicotine Products – please specify) |
| G. | Have you ever swallowed the pouch in whole or in part in the past two weeks? (Yes/No) |
| H. | Did you ever swallow it on purpose? (# of Times) |
| I. | How many times have you swallowed the pouch on purpose in the past two weeks? |
| J. | Did you swallow it in whole or in part? (Whole/In Part) |
| K. | Over the last two weeks, did you spit out your saliva while using the pouch (like people do with chewing tobacco)? |
| L | On how many occasions did you do this? (Every time/most of time that I used [Product], Some of the time that I used [Product], Rarely when I used [Product])? |
