# Supplementary material for: Designing Studies to Inform Tobacco Harm Reduction: Learnings From an Oral Nicotine Pouch Actual Use Pilot Study
Source: JMIR Form Res. 2022 Aug 19;6(8):e37573. doi: 10.2196/37573 (PMC9440415; doi:10.2196/37573)
Supplement: Multimedia Appendix 4 [file formative_v6i8e37573_app4.docx]

Multimedia Appendix 4. Closeout Questionnaire.

|  | Item |
| --- | --- |
| A. | How satisfied are you with your experience of being in this study? (Very satisfied, Satisfied, Neither Satisfied nor dissatisfied, Dissatisfied, Very Dissatisfied, Don’t Know/Unsure, Decline to Answer) |
| B. | Would you recommend joining a research study like this to your family and friends? (Very likely, Likely, Neither likely nor unlikely, Very unlikely, Don’t Know/Unsure, Decline to Answer) |
| C. | Why did you give these ratings? (Verbatim, Decline to Answer) |
| D. | How satisfied were you with each of these study elements?   - Study Introduction and Informed Consent Materials - eDiary - Your compensation for participation - The study team you interacted with   (Very satisfied, Satisfied, Neither Satisfied nor dissatisfied, Dissatisfied, Very Dissatisfied, Don’t Know/Unsure, Decline to Answer) |
| E. | Did you use the Hotline? (Yes, No, Don’t know/Unsure, Decline to Answer) |
| F | How satisfied were you with the Hotline? (Very satisfied, Satisfied, Neither Satisfied nor dissatisfied, Dissatisfied, Very Dissatisfied, Don’t Know/Unsure, Decline to Answer) |
| G. | Why did you give these ratings? (Verbatim, Decline to Answer) |
| H. | Is there anything you would recommend we change for future research like this? (Verbatim, No Suggestions, Decline to Answer) |
